# Using the Novel Mortality-Prevalence Ratio to Evaluate Potentially Undocumented SARS-CoV-2 Infection: Correlational Study

**DOI:** 10.2196/23034

**Published:** 2021-01-27

**Authors:** Sheng-Hsuan Lin, Shih-Chen Fu, Chu-Lan Michael Kao

**Affiliations:** 1 Institute of Statistics National Chiao Tung University Hsinchu Taiwan

**Keywords:** COVID-19, prevalence, mortality, undocumented infection, mortality-prevalence ratio, China

## Abstract

**Background:**

The high prevalence of COVID-19 has resulted in 200,000 deaths as of early 2020. The corresponding mortality rate among different countries and times varies.

**Objective:**

This study aims to investigate the relationship between the mortality rate and prevalence of COVID-19 within a country.

**Methods:**

We collected data from the Johns Hopkins Coronavirus Resource Center. These data included the daily cumulative death count, recovered count, and confirmed count for each country. This study focused on a total of 36 countries with over 10,000 confirmed COVID-19 cases. Mortality was the main outcome and dependent variable, and it was computed by dividing the number of COVID-19 deaths by the number of confirmed cases.

**Results:**

The results of our global panel regression analysis showed that there was a highly significant correlation between prevalence and mortality (ρ=0.8304; *P*<.001). We found that every increment of 1 confirmed COVID-19 case per 1000 individuals led to a 1.29268% increase in mortality, after controlling for country-specific baseline mortality and time-fixed effects. Over 70% of excess mortality could be attributed to prevalence, and the heterogeneity among countries’ mortality-prevalence ratio was significant (*P*<.001). Further, our results showed that China had an abnormally high and significant mortality-prevalence ratio compared to other countries (*P*<.001). This unusual deviation in the mortality-prevalence ratio disappeared with the removal of the data that was collected from China after February 17, 2020. It is worth noting that the prevalence of a disease relies on accurate diagnoses and comprehensive surveillance, which can be difficult to achieve due to practical or political concerns.

**Conclusions:**

The association between COVID-19 mortality and prevalence was observed and quantified as the mortality-prevalence ratio. Our results highlight the importance of constraining disease transmission to decrease mortality rates. The comparison of mortality-prevalence ratios between countries can be a powerful method for detecting, or even quantifying, the proportion of individuals with undocumented SARS-CoV-2 infection.

## Introduction

The first cluster of cases of pneumonia, which was later identified as COVID-19, a disease caused by the SARS-CoV-2 virus [1], was reported in Wuhan, China on December 31, 2019 [2]. The disease outbreak in China eventually developed into a pandemic, which forced widespread changes throughout the world and added substantial disease and economic burden worldwide. As of May 2, 2020, more than 36 countries have reported at least 10,000 cases of COVID-19. A total of around 4 million cases and 274,000 deaths have been reported [2,3]. Numerous studies have been conducted to investigate the biological and epidemiologic characteristics of COVID-19 [4-6]. Most results have been derived from traditional epidemiological models, wherein both COVID-19 mortality (ie, the “case fatality rate” in some literature) and recovery rates were assumed to be constants. However, in a study conducted by Bialek et al [7], heterogeneity in mortality rates was found among countries and cities, but this has been attributed to the assumed underlying medical conditions within an area [8-10]. The trend in mortality over time is also controversial [11-13]. Although results from an exponential growth model have shown an overall exponential decay in mortality within China since the disease outbreak [13], there has been evidence that shows disease prevalence influences disease mortality to a considerable extent. The rapid increase in the number of infections may result in the collapse of the health care system, leading to a sharp rise of mortality [11,12]. Despite the inconsistencies in mortality characteristics between studies, previous analyses have been performed with data that were collected before March, 2020. Up until then, only a few countries reported the number of COVID-19 deaths, whereas most areas were not majorly affected by COVID-19.

This study aims to sophisticatedly quantify the relationship between COVID-19 prevalence and mortality, by using data that have been updated up until May 2, 2020. A linear relationship between prevalence and mortality was observed, and this was referred to as the mortality-prevalence ratio. The global mortality-prevalence ratio was estimated after adjusting for country-specific baseline mortality and time-fixed effects. Country-specific mortality-prevalence ratio values can be used as a powerful index for identifying countries with a substantial number undocumented infections or overburdened health care systems.

## Methods

COVID-19–related data [14] was downloaded from the Johns Hopkins Coronavirus Resource Center. These data included the cumulative number of confirmed cases (C_it_), death cases (D_it_), and recovered cases (R_it_) of the i^th^ country from January 22 to May 2, 2020. We then matched each country with their respective national population data, which were provided by World Population Review [15]. Countries without a matched population were excluded from this study. After exclusion, 174 countries remained in our dataset. We later aggregated the remaining countries to obtain the corresponding global counts.

For each country and each time point, we computed the following 3 metrics, along with the global data: (1) the number of cases still in treatment (CT_it_), which represents the total number of COVID-19 cases that involved medical assistance at time t; (2) the prevalence of COVID-19 in country i at time t (P_it_); and (3) COVID-19 mortality in country i at time t (M_it_). For the sake of model stability, the analyses were only performed on countries with a C_it_ of ≥10,000. The following equations were used to calculate each metric:

CT_it_ = C_it_ – D_it_ − R_it_ .....**(1)**

P_it_ = C_it_/total population of country i .....**(2)**

M_it_ = D_it_/C_it_ .....**(3)**

To investigate the association between mortality and prevalence after adjusting for the baseline mortality in each country and the effect of regular fluctuation over time, we built the following panel regression model (ie, Model 1):

M_it_ = β_country_ + β_t_ + γP_it_ + ε_it_ .....**(4)**

In this model, β_country_ represents the country-specific baseline mortality; β_t_ is the time-fixed effect on the mortality; γ represents the global association between P_it_ and M_it_, which we referred to as the global mortality-prevalence ratio; and ε_it_ is the residual. To meet the assumption that the mortality-prevalence ratio varies in each country, we built a panel regression model (ie, Model 2), in which the global mortality-prevalence ratio was replaced with the country-specific mortality-prevalence ratio, γ_country_. Model 2 is described as follows:

M_it_ = β_country_ + β_t_ + γ_country_P_it_ + ε_it_ .....**(5)**

In this model, γ_country_ is the country-specific association between P_it_ and M_it_, which we referred to as the country-specific mortality-prevalence ratio. Furthermore, we tested whether γ_country_ differed between each country with an analysis of variance test. We also tested whether the difference could be treated as the random effect of a normal population with the Shapiro-Wilk normality test. All analyses were conducted with R version 3.5.2. The approval of an institutional review board was not required because no individual-level/personal data were used.

## Results

**Figure 1:**
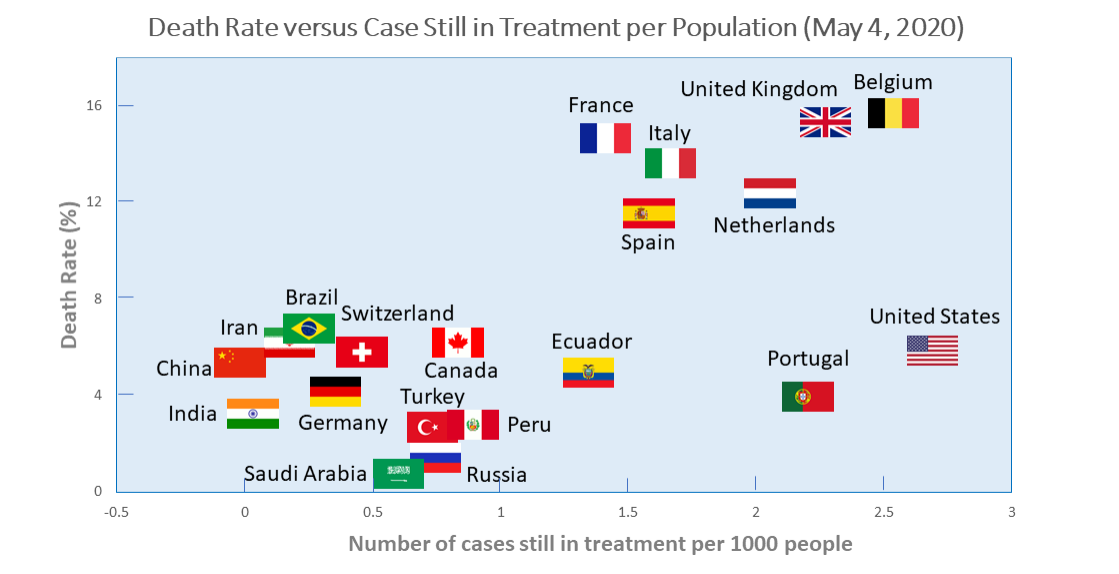
COVID-19 mortality and prevalence of all countries (ρ=0.8304; *P*<.001). Only the top 20 countries with the highest prevalence are shown.

Table 1 shows the population and the total number of confirmed cases, death cases, and recovered cases for countries that reported at least 10,000 confirmed cases by May 2, 2020. Figure 1 shows the association between COVID-19 prevalence and mortality among these countries. The Spearman correlation coefficient was 0.8304 (*P*<.001) and the Pearson correlation coefficient was 0.3385 (*P*=.04). These values indicated a significant positive correlation between prevalence and mortality. COVID-19 mortality and prevalence were relatively high in the United Kingdom and Belgium, while the United States had a high prevalence and a relatively low mortality compared to countries with similar prevalence levels, such as China and Canada.

It is worth mentioning that the positive correlation between mortality and prevalence is not restricted to COVID-19. For example, when considering the prevalence and mortality of severe acute respiratory syndrome (SARS) on July 31, 2003 based on data from the World Health Organization, the Spearman correlation coefficient was 0.3915 (*P*=.03). Since the number of countries involved with the COVID-19 pandemic is considerably larger than those involved with the SARS pandemic, the correlation between COVID-19 mortality and prevalence is statistically more significant than the correlation between SARS mortality and prevalence.

**Figure 2:**
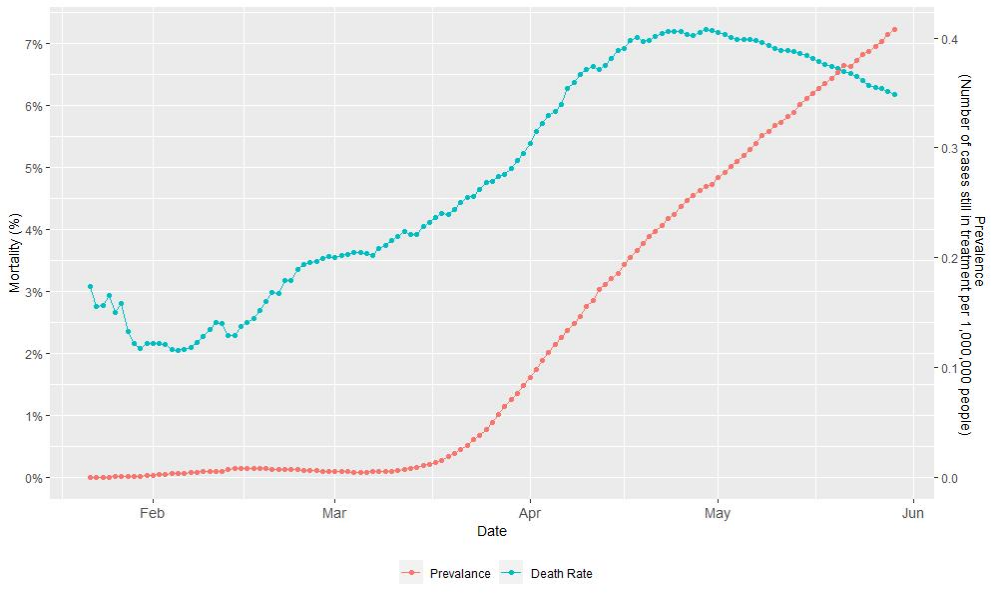
Trends of global COVID-19 mortality and prevalence over time.

The relationship between global COVID-19 prevalence and mortality can also be observed when time is considered (Figure 2). Both prevalence and mortality increased over time.

**Table 1:** Total population and the total number of confirmed cases, death cases, and recovered cases for countries that reported at least 10,000 confirmed cases by May 2, 2020.

Country	Total population, N	Confirmed cases, n	Deaths, n	Recovered cases, n
Austria	9,006,398	15,558	596	13,180
Belarus	9,449,323	15,828	97	3117
Belgium	11,589,623	49,517	7765	12,211
Brazil	212,559,417	97,100	6761	40,937
Canada	37,742,154	57,926	3684	23,814
Chile	19,116,201	18,435	247	9572
China	1,439,323,776	83,959	4637	78,586
Ecuador	17,643,054	27,464	1371	2132
France	65,273,511	168,518	24,763	50,663
Germany	83,783,942	164,967	6812	129,000
India	1,380,004,385	39,699	1323	10,819
Indonesia	273,523,615	10,843	831	1665
Iran	83,992,949	96,448	6156	77,350
Ireland	4,937,786	21,176	1286	13,386
Israel	8,655,535	16,185	229	9593
Italy	60,461,826	209,328	28,710	79,914
Japan	126,476,461	14,571	474	3205
Mexico	128,932,753	22,088	2061	12,377
Netherlands	17,134,872	40,434	5003	138
Pakistan	220,892,340	19,103	440	4817
Peru	32,971,854	42,534	1200	12,434
Poland	37,846,611	13,375	664	3762
Portugal	10,196,709	25,190	1023	1671
Qatar	2,881,053	14,872	12	1534
Romania	19,237,691	12,732	771	4547
Russia	145,934,462	124,054	1222	15,013
Saudi Arabia	34,813,871	25,459	176	3765
Singapore	5,850,342	17,548	17	1347
Spain	46,754,778	216,582	25,100	117,248
Sweden	10,099,265	22,082	2669	1005
Switzerland	8,654,622	29,817	1762	24,200
Turkey	84,339,067	124,375	3336	58,259
Ukraine	43,733,762	11,411	279	1498
United Arab Emirates	9,890,402	13,599	119	2664
United Kingdom	67,886,011	183,500	28,205	896
United States	331,002,651	1,132,539	66,369	175,382

In order to sophisticatedly estimate the relationship between mortality and prevalence, time and country-specific baseline mortalities in Model 1 were adjusted. The estimations for all coefficients are shown in Table 2. The global mortality-prevalence ratio, which was represented by γ in Model 1, was estimated to be 12.9268 (*P*<.001). This number can be interpreted as follows: an increment of 1 COVID-19 case per 1000 people is coupled with a 1.29268% (ie, 12.9268 × 1/1000 × 100) increase in mortality. The R^2^ value that was calculated from Model 1 was 98.11%, and the partial R^2^ value for was 70.41%. These values indicated that COVID-19 prevalence could roughly explain the 70% heterogeneity in excess mortality after controlling for country-specific baseline mortality and time-fixed effects. The analysis of variance test showed potential heterogeneity in the mortality-prevalence ratios among different countries (*P*<.001). Therefore, we performed a panel regression analysis based on Model 2, as shown in Table 2. It should be noted that the partial R^2^ value for the mortality-prevalence ratio increased to 89.37% in Model 2.

**Table 2:** Estimation of all coefficients for Model 1 and Model 2.

Model				Estimation	*P* value	Partial R^2^
**Model 1^a^**						
	Mortality-prevalence ratio (ie, γ)			12.9268	<.001	0.7041
**Model 2^b^**						
	**Country-specific mortality-prevalence ratio (ie, γ_country_)**					0.8937
		**All data**				
			Austria	–30.8171	<.001	
			Belarus	–19.2428	.27	
			Belgium	45.4706	<.001	
			Brazil	71.4636	.002	
			Canada	65.5696	<.001	
			Chile	–27.4605	.47	
			China	347.7652	<.001	
			Ecuador	–33.2373	<.001	
			France	43.1863	<.001	
			Germany	–22.7914	<.001	
			India	–341.5505	.34	
			Indonesia	–1205.3198	.79	
			Iran	–52.9484	<.001	
			Ireland	–23.1711	<.001	
			Israel	–14.2313	.22	
			Italy	13.0634	<.001	
			Japan	334.2415	.17	
			Mexico	–42.3179	.79	
			Netherlands	4.1811	.08	
			Pakistan	11.9388	.96	
			Peru	–9.3371	.16	
			Poland	286.3706	.28	
			Portugal	–5.4107	.10	
			Qatar	–14.4505	.006	
			Romania	81.1284	.65	
			Russia	–14.0904	.03	
			Saudi Arabia	–26.3058	.09	
			Singapore	–14.4186	.01	
			Spain	7.1163	<.001	
			Sweden	23.1690	<.001	
			Switzerland	–32.3043	<.001	
			Turkey	–17.0113	<.001	
			Ukraine	–82.0183	.88	
			United Arab Emirates	–14.6387	.60	
			United Kingdom	14.4444	<.001	
			United States	–4.1818	<.001	
		**All data excluding those collected from China after February 17, 2020**				
			Austria	–18.4144	<.001	
			Belarus	–5.9724	.73	
			Belgium	58.4075	<.001	
			Brazil	89.1904	<.001	
			Canada	80.0715	<.001	
			Chile	–12.7334	.73	
			China	–1.6094	.99	
			Ecuador	–20.0146	<.001	
			France	53.6918	<.001	
			Germany	–17.5007	.001	
			India	–265.0415	.46	
			Indonesia	–1179.8199	.79	
			Iran	–62.5286	<.001	
			Ireland	–10.4239	<.001	
			Israel	–3.2505	.78	
			Italy	20.4366	<.001	
			Japan	359.2586	.14	
			Mexico	–31.1910	.85	
			Netherlands	17.1263	<.001	
			Pakistan	33.3642	.88	
			Peru	–4.5690	.48	
			Poland	308.5810	.24	
			Portugal	8.3993	.01	
			Qatar	–1.3688	.79	
			Romania	98.2757	.58	
			Russia	0.4826	.94	
			Saudi Arabia	–12.5797	.42	
			Singapore	–1.2272	.81	
			Spain	16.7545	<.001	
			Sweden	37.2237	<.001	
			Switzerland	–19.2162	<.001	
			Turkey	–3.9981	.34	
			Ukraine	–67.5598	.90	
			United Arab Emirates	–1.1999	.97	
			United Kingdom	27.0183	<.001	
			United States	7.5391	<.001	

^a^The R^2^ value for Model 1 was 0.9811 (*P*<.001).

^b^The R^2^ value for Model 2 was 0.9931 (*P*<.001).

**Figure 3:**
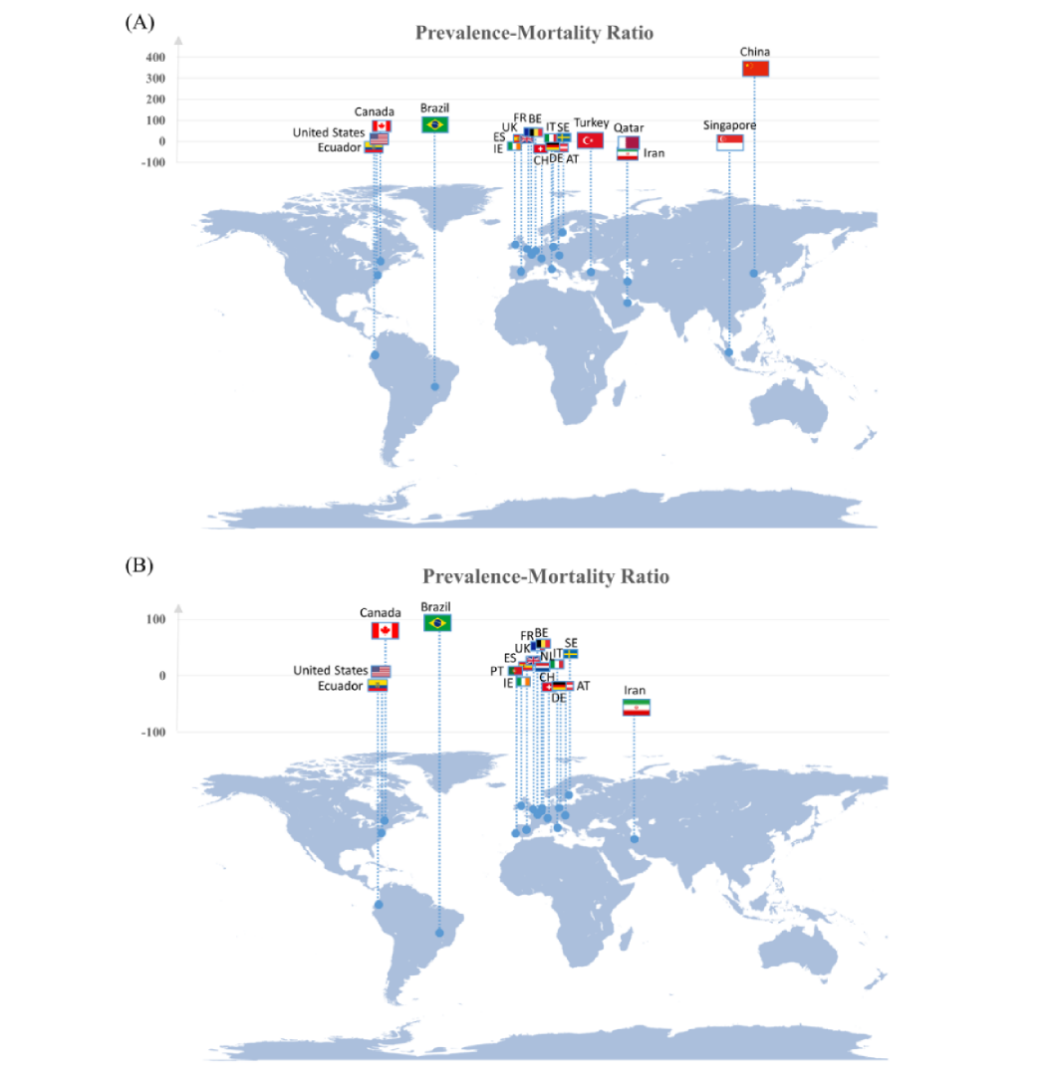
Countries with significant country-specific mortality-prevalence ratios based on (A) all data and (B) all data excluding those collected from China after February 17, 2020.

**Figure 4:**
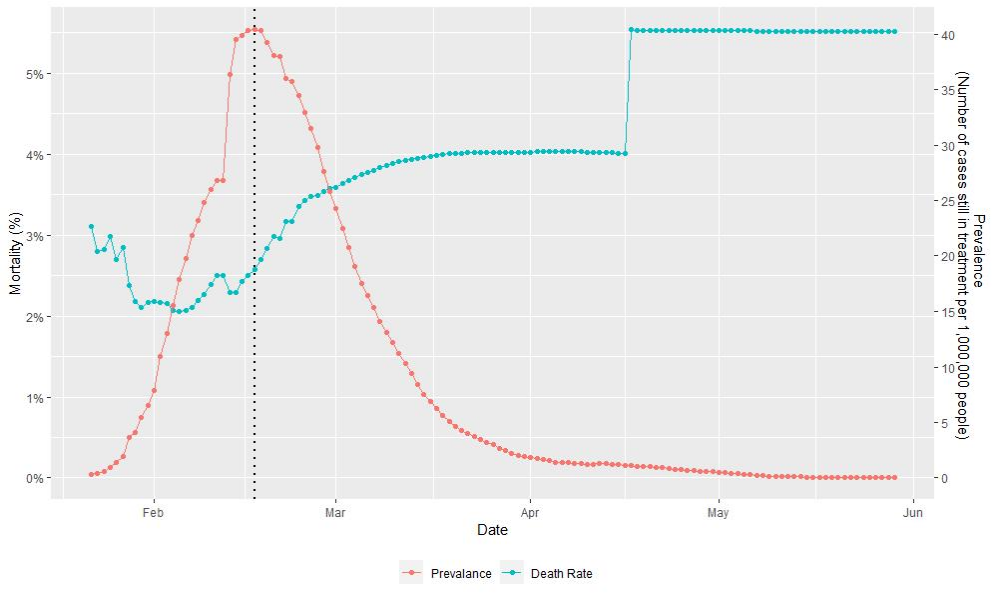
COVID-19 prevalence and mortality reported by China over time.

We obtained estimated country-specific mortality-prevalence ratios that ranged from −1205 to 348 from the 36 countries that were included in our analysis (Figure 3). Absolute mortality-prevalence ratio values of >100 were found in 5 countries (ie, Indonesia, India, Poland, Japan, and China), of which China was the only country that had a significantly different mortality-prevalence ratio (348; *P*<.001). The results of our Shapiro-Wilk normality test meant that we could reject the hypothesis that all significant country-specific mortality-prevalence ratios came from a normal distribution (*P*<.001). As we further investigated the pattern of China’s mortality-prevalence ratio over time, we noted that the correlation had turned from positive to negative after February 17, 2020 (Figure 4). This disparity was not observed if the data that was collected after February 17, 2020 was excluded (Figure 3) (Shapiro-Wilk normality test: *P*=.78).

## Discussion

This is the first study to assess the correlation between COVID-19 prevalence and mortality after adjusting for time-fixed effects and country-specific baseline mortality. We proposed the mortality-prevalence ratio as a novel characteristic for an infectious disease pandemic because of the high association between disease mortality and prevalence. In addition, a disparity in the mortality-prevalence ratios of 5 countries was observed; China was the only country with a significant mortality-prevalence ratio (348; *P*<.001). The disparity of China’s mortality-prevalence ratio was due to the data reported after February 17, 2020. Although the mortality was proportional to the prevalence, the mortality-prevalence ratio was relatively robust to changes in prevalence (Figure 3). A high peak in mortality-prevalence ratios could be explained by a high proportion of undocumented infections within a country, which might be attributed to the limited number of diagnostic kits or changes in surveillance policies. An alternative explanation for the sudden rise of mortality could be that the health care system in China was relatively weak after February 17. However, this argument contradicts the fact that China’s overall baseline country-specific mortality was typically followed by a steady increase in disease prevalence after February 17. The evolution of the pathogenicity and transmissibility of SARS-CoV-2 within China during this period could be another alternative reason for the disparity in mortality-prevalence ratios. Further studies are required to determine the underlying cause of this sharp increase in the mortality-prevalence ratio.

This study revealed the importance of public policies that aim to prevent disease transmission. These policies include social distancing, restricting travel, encouraging the wearing of facial masks and hand washing, and cancelling large events. Although the mortality rate of a certain infectious disease is traditionally assumed to be a constant in an infectious dynamic model [16], it is conceivable that a highly infectious disease affects the quality and availability of a health care system. The fast consumption of ventilation machines and the decline of nurse-to-patient ratios accelerate mortality. Prevention policies not only lower the financial burden on COVID-19 diagnosis and treatment, but also reduce COVID-19 mortality. Therefore, when future cost-effectiveness analyses are performed with respect to the balance between economic recovery and public health, it is crucial to consider the positive association between disease prevalence and mortality and the costs that come with it.

There are several limitations in this study. First, all results were based on ecological and panel data. Such data lack individual-level information. Therefore, the ecological fallacy would occur when trying to infer causality at the individual level [17]. The temporal effects of prevalence on mortality should also be confirmed to verify country-level causality. Second, although the prevalence of COVID-19 can generally be interpreted as an acute burden of health care, this relationship can be better verified when data on the actual insufficiencies of health care systems are available. Third, disease prevalence relies on accurate diagnoses and comprehensive surveillance, which can be difficult to achieve due to practical or political concerns. This was especially true at the beginning of the COVID-19 pandemic, which was when tests for COVID-19 were not accurate and data on people who died from COVID-19 may not have been captured. In this study, although countries with undocumented infections can be partially inferred with disparities in mortality-prevalence ratios, a more direct index merits further study.

In conclusion, we observed the relationship between COVID-19 mortality and prevalence and quantified this relationship as mortality-prevalence ratios. Our results highlight the benefit of constraining disease transmission to reduce mortality. Disparities in mortality-prevalence ratios can also be a powerful tool to detect, or even quantify, the proportion of undocumented infections.

## Abbreviations

SARS: severe acute respiratory syndrome
